# Ambient Air Pollution and Atherosclerosis in Los Angeles

**DOI:** 10.1289/ehp.7523

**Published:** 2004-11-22

**Authors:** Nino Künzli, Michael Jerrett, Wendy J. Mack, Bernardo Beckerman, Laurie LaBree, Frank Gilliland, Duncan Thomas, John Peters, Howard N. Hodis

**Affiliations:** Divisions of Environmental Health and Biostatistics, Department of Preventive Medicine, and Atherosclerosis Research Unit, Division of Cardiovascular Medicine, Keck School of Medicine, University of Southern California, Los Angeles, California, USA

**Keywords:** air pollution, atherosclerosis, particulate matter

## Abstract

Associations have been found between long-term exposure to ambient air pollution and cardiovascular morbidity and mortality. The contribution of air pollution to atherosclerosis that underlies many cardiovascular diseases has not been investigated. Animal data suggest that ambient particulate matter (PM) may contribute to atherogenesis. We used data on 798 participants from two clinical trials to investigate the association between atherosclerosis and long-term exposure to ambient PM up to 2.5 μm in aerodynamic diameter (PM_2.5_). Baseline data included assessment of the carotid intima-media thickness (CIMT), a measure of subclinical atherosclerosis. We geocoded subjects’ residential areas to assign annual mean concentrations of ambient PM_2.5_. Exposure values were assigned from a PM_2.5_ surface derived from a geostatistical model. Individually assigned annual mean PM_2.5_ concentrations ranged from 5.2 to 26.9 μg/m3 (mean, 20.3). For a cross-sectional exposure contrast of 10 μg/m3 PM_2.5_, CIMT increased by 5.9% (95% confidence interval, 1–11%). Adjustment for age reduced the coefficients, but further adjustment for covariates indicated robust estimates in the range of 3.9–4.3% (*p*-values, 0.05–0.1). Among older subjects (≥60 years of age), women, never smokers, and those reporting lipid-lowering treatment at baseline, the associations of PM_2.5_ and CIMT were larger with the strongest associations in women ≥60 years of age (15.7%, 5.7–26.6%). These results represent the first epidemiologic evidence of an association between atherosclerosis and ambient air pollution. Given the leading role of cardiovascular disease as a cause of death and the large populations exposed to ambient PM_2.5_, these findings may be important and need further confirmation.

A large body of epidemiologic evidence suggests associations between ambient air pollution and cardiovascular mortality and morbidity (Peters and Pope 2002; Pope et al. 2004). All of these studies focus on events occurring at a late stage of vascular disease processes. The impact of air pollution on the underlying preclinical conditions remains poorly understood. We hypothesize that current levels of ambient particulate matter (PM) up to 2.5 μm in aerodynamic diameter (PM_2.5_) may contribute to atherosclerosis, leading to subclinical anatomical changes that play a major role in cardiovascular morbidity and mortality later in life. Animal studies support our hypothesis by showing that inhalation of ambient PM promotes oxidative lung damage, including alveolar and systemic inflammatory responses (Becker et al. 1996; Dye et al. 2001; Fujii et al. 2002; Goto et al. 2004; Suwa et al. 2002; van Eeden et al. 2001).

We investigated the association between residential ambient PM2.5 and carotid artery intima-media thickness (CIMT) using pre-randomization baseline data from two recent clinical trials conducted in Los Angeles, California (Hodis et al. 2002). CIMT is a well-established quantitative measure of generalized atherosclerosis that correlates well with all of the major cardiovascular risk factors, with coronary artery atherosclerosis, and with clinical cardiovascular events (Mack et al. 2000). It is an established tool for investigating the contribution of long-term exposures such as smoking or passive smoking to subclinical stages of atherosclerosis at any given age (Diez-Roux et al. 1995; Howard et al. 1994, 1998). This is the first study to assess the association of atherosclerosis with air pollution.

## Materials and Methods

### Population and health assessment

We used baseline health data from two randomized, double-blind, placebo-controlled clinical trials conducted at the University of Southern California Atherosclerosis Research Unit (Hodis et al. 2002). The Vitamin E Atherosclerosis Progression Study (VEAPS) investigated the effects of vitamin E on the progression of atherosclerosis measured by CIMT. The B-Vitamin Atherosclerosis Intervention Trial (BVAIT) focused on the effect of vitamin B supplements on the progression of atherosclerosis (trial in progress). Baseline assessment in both trials included CIMT measured between 1998 and 2003 using the same standardized methods (Hodis et al. 2002; Selzer et al. 1994, 2001). Recruitment of volunteers occurred over the entire Los Angeles Basin, covering a geographic area of approximately 64,000 km^2^.

Eligible subjects for the VEAPS trial (*n* = 353) were men and women ≥40 years of age with slightly increased LDL cholesterol (≥3.37 mmol/L) but with no clinical signs or symptoms of cardiovascular disease (CVD) (Hodis et al. 2002). Subjects with diabetes, diastolic blood pressure > 100 mm Hg, thyroid disease, serum creatinine > 0.065 mmol/L, life-threatening diseases, or high alcohol intake were excluded.

BVAIT (*n* = 506) had a similar design to that of VEAPS. Men and women > 40 years of age were prescreened to meet study criteria (fasting plasma homocysteine ≥8.5 μmol/L; postmenopausal for women; no evidence of diabetes, heart disease, stroke, or cancer). Subjects were excluded on the basis of any clinical signs or symptoms of CVD, diabetes or fasting serum glucose ≥140 mg/dL, triglyceride levels ≥150 mg/dL, serum creatinine > 1.6 mg/dL, high blood pressure, untreated thyroid disease, life-threatening disease with prognosis < 5 years, or high alcohol intake.

Thus, our study included “healthy” subjects with biomarkers (elevated LDL cholesterol or homocysteine) that suggested an increased risk of future CVDs (*n* = 859). Fifty-eight subjects were excluded in the exposure assignment process because they lived outside the area with PM_2.5_ data. Three subjects had missing data in at least one of the covariates used in the models. Our total sample consisted of 798 participants.

### Health measures, including CIMT

Our main outcome of interest is CIMT. In both trials, high-resolution B-mode ultrasound images of the right common carotid artery were obtained before the intervention (baseline) with a 7.5-MHz linear array transducer attached to an ATL Ultramark-4 Plus Ultrasound System (Ultramark, Bothell, WA). We used this baseline CIMT measurement as the outcome. Details of this highly reproducible method are published (Hodis et al. 2002; Selzer et al. 1994, 2001). Blood pressure, height, and weight were measured with standard procedures.

The baseline questionnaires included an assessment of all major CVD risk factors and covariates, including clinical events, diet, use of prescription medications, physical activity, current and past smoking and passive smoking, and vitamin supplements. Age, education, and other sociodemographic factors were available for each subject. Fasting blood samples were also drawn for lipid measurements. Data used in our analyses were collected with the same tools in both trials.

### Exposure assignment

**Figure 1:**
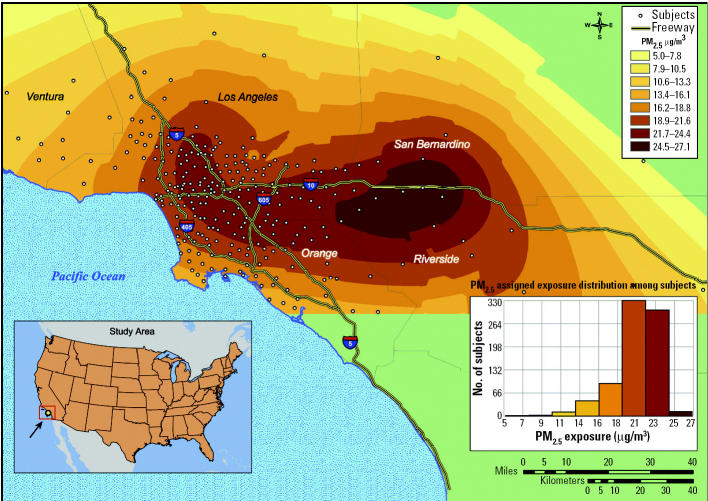
ZIP code locations of the study population geocoded on the PM_2.5_ surface, modeled with 2000 PM_2.5_ data, and distribution of individually assigned concentrations.

To assess exposure we chose a novel approach derived from a geographic information system (GIS) and geostatistics. This method allows for assignment of long-term mean ambient concentrations of PM_2.5_ to the ZIP code area of each subject’s residential address (Künzli and Tager 2000). The resulting surface of PM_2.5_ covered the entire Los Angeles metropolitan area. The surface is derived from a geostatistical model and data from 23 state and local district monitoring stations (during 2000). These monitors are located across the Los Angeles region to characterize urban levels of pollution. To assign exposure, PM_2.5_ data were interpolated using a combination of a universal kriging model with a quadratic drift and a multi-quadric radial basis function model (Bailey and Gatrell 1995; Burrough and McDonnell 1998). We averaged the two surfaces based on 25-m grid cells. Examination of errors from the universal model showed that > 50% of the study area had assigned values within 15% of monitored concentrations, whereas 67% were within 20%. The larger errors were on the periphery of our study area, where the density of study participants was the lowest. We linked the ZIP code centroids of each subject with the exposure surface through a geocoding database [Environmental Systems Research Institute (ESRI) 2004]. Figure 1 illustrates the PM_2.5_ surface with the geolocated ZIP codes. Individually assigned PM_2.5_ data had a range from 5.2 to 26.9 μg/m^3^ (mean, 20.3), thus exceeding the range observed across 156 metropolitan areas used in the largest cohort study of air pollution and mortality (Pope et al. 2002). All models were implemented with ArcScript from ESRI (Redlands, CA).

### Statistical analyses

We tested the univariate and multivariate associations between CIMT and ambient PM_2.5_ using linear regression analyses. Extensive residual diagnostics indicated some heteroskedasticity, which was rectified with the natural log-transformed CIMT. We adjusted for factors that were statistically associated with both CIMT and ambient PM_2.5_ (age, male sex, low education, and low income). Next, we expanded the models using covariates that were associated with either PM_2.5_ or CIMT, including indicator variables for current second-hand smoke exposure and current and former personal smoking. We then added covariates that play a role in atherosclerosis such as blood pressure, LDL cholesterol, or proxy measures such as reporting treatment with antihypertensives or lipid-lowering medications at study entry. These factors may affect the pathophysiologic pathways linking air pollution exposure and atherosclerosis (Ross 1999); thus, such models may overadjust the coefficients. We chose this conservative approach to test the sensitivity of the effect estimates under a broad range of model assumptions.

There is increasing evidence that host factors such as age, sex, or underlying disease and risk profiles may modify the effects of air pollution (Pope et al. 2002; Zanobetti and Schwartz 2002). Furthermore, the finding of atherosclerosis in PM-exposed rabbits was based on a hyperlipidemic trait (Suwa et al. 2002). Therefore, we also stratified by sex, age (< 60 years, ≥60 years), smoking status, and lipid-lowering drug therapy.

## Results

**Figure 2:**
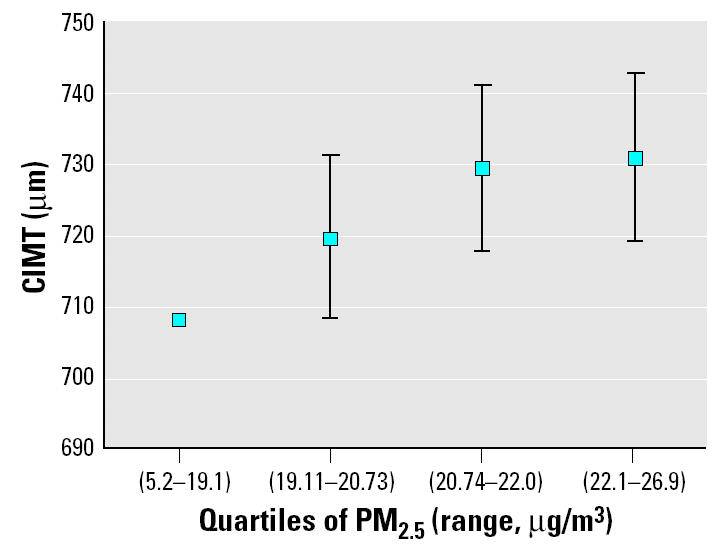
Mean CIMT ± 1 SE among quartiles of the PM_2.5_ distribution. The *y*-axis shows mean CIMT levels at the population average of the adjustment covariates (age, sex, education, and income). The first quartile is the reference group.

Table 1 summarizes the main characteristics of the study population and among main subgroups. Table 2 presents the percent change in CIMT in association with a 10 μg/m^3^ contrast in ambient PM_2.5_ concentrations for three cross-sectional regression models. The unadjusted model indicates a 5.9% [95% confidence interval (CI), 1–11%] increase in CIMT per 10 μg/m3 PM_2.5_. For the observed contrast between lowest and highest exposure (20 μg/m^3^ PM_2.5_), this corresponds to a 12.1% (2.0–23.1%) increase in CIMT. The only covariate with a substantial effect on the point estimate was age, which reduced the effect from 5.9 to 4.3% (0.4–9%) per 10 μg/m3 PM_2.5_. This change agrees with the age-related effect modification. Otherwise, effect estimates across the models remained robust, in the range of 3.9–4.3% with *p*-values from 0.05 to 0.1. To corroborate the exposure–response relationship, we also categorized PM_2.5_ levels into quartiles. Figure 2 shows the adjusted mean CIMT across these four groups of equal sample size at the mean levels of the covariates (age, sex, education, and income). The trend across the exposure groups was statistically significant (*p* = 0.041). The unadjusted means of CIMT among these quartiles of exposure were 734, 753, 758, and 774 μm, respectively.

**Figure 3:**
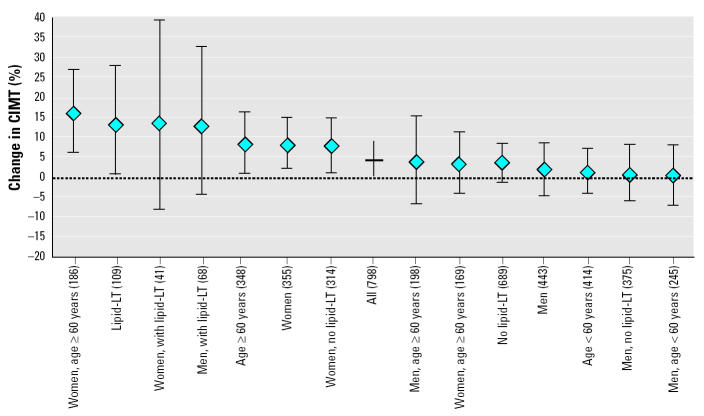
Percent difference and 95% CI in CIMT associated with a 10 μg/m^3^ contrast in ambient PM_2.5_ in all subjects and in subgroups. Lipid-LT, lipid-lowering therapy. All estimates are based on the cross-sectional linear model with log intima-media thickness as the dependent variable and home outdoor PM_2.5_ as the independent variable, adjusted for sex, age, education, and income. Numbers in parentheses are numbers of subjects per group. Data are ordered by size of point estimate; the null effect line is indicated by a dash.

The associations between CIMT and PM_2.5_ were substantially stronger among 109 subjects reporting lipid-lowering medication at study entry, both in men and in women (Table 2, Figure 3). The crude effect reached 15.8% (2–31%) per 10 μg/m^3^ PM_2.5_, with adjusted values ranging between 12 and 16%. Despite the small sample size, *p*-values of all models were mostly < 0.1 and often < 0.05.

Results also suggest significant age and sex interactions, with much larger effects in women and in the older age group (Figure 3). Effect estimates in women were statistically significant and typically in the range of 6–9% per 10 μg/m3 PM_2.5_. Associations were strongest among women ≥60 years of age (*n* = 186), leading to crude estimates of 19.2% (9–31%). Adjusted coefficients ranged from 14 to 19%, being statistically significant in all models and sensitivity analyses.

Among never smokers (*n* = 502), the effect estimate reached 6.6% (1.0–12.3%). The estimate was small and not significant in current (*n* = 30) and former smokers (*n* = 265).

## Discussion

Our study presents the first evidence for an association between CIMT and long-term exposure to ambient air pollution. As recently reviewed in a statement of the American Heart Association (Brook et al. 2004) substantial epidemiologic and experimental evidence suggests a contribution of ambient air pollutants on cardiovascular mortality and morbidity. However, these studies focus on acute and subacute effects on cardiac autonomic function, inflammatory or thrombogenic markers, arrhythmia, myocardial infarction, cardiovascular hospital admission, and death. The only outcome considered in long-term air pollution studies has been mortality. The relative risks for acute effects on mortality have been substantially smaller than those observed for long-term associations (Pope et al. 2002; Samet et al. 2000). As shown previously, cohort studies are capable of capturing acute and chronic effects of air pollution on the course of diseases that ultimately lead to premature death (Künzli et al. 2001). In contrast, time-series and panel studies investigate only the associations of event occurrence with the most recent exposure (Künzli et al. 2001). Thus, if air pollution has both acute and cumulative long-term effects, one expects larger mortality coefficients in cohort studies. CIMT reflects long-term past exposure; thus, we provide the first evidence for chronic effects of air pollution on atherogenesis that may in part explain the above mentioned discrepancy between acute and long-term risk estimates (Pope et al. 2002; Samet et al. 2000).

There are several major aspects to be considered in the interpretation of this new finding, mainly the strength in the exposure assignment, the limited evidence for bias, the differences in effects within subgroups, and plausibility.

### Exposure assignment

The individual residence-based assignment of exposure represents a substantial improvement over most studies that have relied on central monitors or on binary road buffers combined with basic interpolation (Hoek et al. 2002; Pope et al. 2004). As a sensitivity analysis, we used weighted least-squares models with the weights specified as the inverse of the standard errors from the universal kriging model to down-weight estimates with larger error. In addition, we implemented models based solely on the universal kriging estimate. In both instances results were robust and similar to what we found with our main model.

Time–activity studies show that people spend most of their time in or around home, and our restriction of exposure assessment on residential address captures the most relevant part of exposure (Leech et al. 2002). PM_2.5_ generally displays spatially homogeneous distributions across small areas such as neighborhoods and blocks, and as a result, the ambient conditions at the ZIP code centroid likely reflect the levels expected at home outdoors (Roosli et al. 2000). PM_2.5_ of outdoor origin will also penetrate indoors, and correlations between long-term outdoor PM concentrations and indoor levels of PM from outdoor origin is high (Sarnat et al. 2000). Exposure to ambient air pollution while working and during commute are not included in our exposure term but are considered to be a relevant source of exposure (Riediker et al. 2003). Although most likely a random misclassification with biases toward the null, the errors may affect subgroups differently, thus explaining part of the observed interactions.

In Los Angeles, no clear trends have been observed in PM_2.5_ concentrations over the past 5–10 years. The year 2000 surface characterizes the prevailing mean PM_2.5_ concentrations across several years and can be considered a measure of long-term past exposure. This year also sits in the middle of the baseline recruitment period. Overall, the various limitations in our exposure assignment may add some random error, biasing results toward weaker associations (Thomas et al. 1993).

We also assigned ambient ozone to ZIP code centroids. Inclusion of ozone in the models had no impact on the PM_2.5_ coefficients or the SEs. Ozone and PM_2.5_ were not correlated (*r* = –0.17), and the PM_2.5_ estimates were not substantially different in low-and high-ozone regions. The estimates of association for ozone were positive but not statistically significant and much smaller than for PM_2.5_. This finding must be put in context of the specific challenges in determining long-term exposure to ozone, which are substantially different than in the case of PM exposure. In contrast to PM_2.5_ from outdoor origin, ambient ozone levels have lower correlations with personal exposure (Avol et al. 1998; Sarnat et al. 2000, 2002); therefore, the ability to detect effects of ozone will likely be reduced due to greater misclassification.

### Biases

Our subjects were a nonrandom sample of “healthy” volunteers with above-average education, meeting strict inclusion criteria for the two clinical trials. Although we cannot exclude some systematic selection biases affecting the cross-sectional data, it is unlikely that subjects with preclinical signs of atherosclerosis would have been more likely to volunteer if they lived in more polluted areas. Although the selection of subjects limits the generalization to other populations, we do not expect this to lead to over- or underestimating the cross-sectional associations. The two trials recruited subjects independently; thus, the effects may be compared across trials to evaluate the potential influence of selecting volunteers. The populations differed with regard to age, smoking habits, baseline LDL and treatment, blood pressure, active and passive smoking, and other relevant factors; thus, the PM_2.5_ coefficients were smaller and were not statistically significant in the VEAPS trial with its younger population. However, after taking these factors into account, the associations with ambient PM_2.5_ were similar. For example, among elderly women of VEAPS (*n* = 70) and BVAIT (*n* = 116), the effect estimate was 18.1% (–0.1 to 36.3.%) and 13.6% (2.8–24.4.%), respectively. There is some evidence for larger effects in subjects with cardiovascular risk factors, indicated by prescriptions of lipid-lowering treatment. Our trials excluded subjects with clinically manifest CVDs. Moreover, if air pollution amplifies systemic inflammation among those prone to atherosclerosis, exclusion of subjects with high LDL may be a source of bias. One may expect effect estimates in a less selected, less healthy population to be larger than those reported.

The wealth of baseline data from these clinical trials offered the opportunity to control for a broad array of covariates. Apart from the effect of age adjustment, estimates were robust to numerous combinations of covariates, including income, education, active and passive tobacco smoke, cardiovascular prescriptions, vitamin intake, and physical activity. Uncontrolled or residual confounding appears to be an unlikely explanation for these results. Among women, adjustment for hormone replacement therapies did not affect the PM_2.5_ estimates.

In previous studies, we found that spatial autocorrelation in the residuals could affect the size and significance of pollution coefficients (Jerrett et al. 2003a). We investigated spatial autocorrelation of the unstandardized residuals. We assessed autocorrelation with first-order, adjusted first-order, and second-order spatial weight matrices based on nearest neighbor contiguity, but we found no evidence of spatial autocorrelation. This supports the conclusion that the models supply efficient unbiased estimates (Jerrett et al. 2003b). As part of our sensitivity analyses, we also derived PM_2.5_ surfaces using different interpolations and weighted least squares with weights equal to the inverse of the standard error of the exposure estimate. All approaches produced very similar results.

### Evidence for effect modification

The data suggest substantial interactions with age, sex, smoking, and underlying cardiovascular risk factors. Given the reduced sample size among subgroups, the recruitment of volunteers, and the cross-sectional nature of the data, it is difficult to fully explore the causes of the observed modifications of associations and to establish susceptibility profiles. If the exposure misclassifications differed across subgroups, part of the interactions may be explained by differential exposure error. The sex and age difference could also be an artifact due to measurement error in the assigned exposure because time spent in commuting and location of work places may be different in men and women and in the young and elderly. Empirical studies on mobility suggest women have smaller activity spaces than men and younger groups, meaning they tend to spend more time in and around the home (Kwan and Lee 2004), and the same is probably true of the elderly compared with younger groups. Exposure measurement error may be reduced in those spending more time at home, leading to stronger effects (Thomas et al. 1993). Moreover, differences in statistical power may play a role as well; as shown at least for the 25–40-year age range, power to detect effects on CIMT is larger in women than in men (Stein et al. 2004).

The finding that those reporting prescriptions of lipid-lowering medications at baseline showed stronger associations of CIMT with PM_2.5_ merits further investigation. This result agrees with the observed effects of PM on atherosclerosis in experiments conducted in hyperlipidemic rabbits (Goto et al. 2004; Suwa et al. 2002). The systemic inflammatory and atherogenic reaction in these rabbits was related to the amount of PM contained in the alveolar macrophages. In our study, being under lipid-lowering therapy is an indicator for risk profiles prone to atherogenesis. Those subjects were mostly men (64%) and, on average, older, more often active or passive smokers, and almost twice as likely to report antihypertensive treatment. The systemic response to ambient PM may amplify and expand the oxidation of LDL cholesterol among these susceptible subjects, consequently contributing to injury in the artery wall (Goto et al. 2004; Ross 1999). Investigations of short-term effects of ambient air pollution on mortality also suggest that underlying risk profiles such as diabetes may amplify susceptibility to ambient PM (Zanobetti and Schwartz 2002), and similar findings have been shown with smoking and diabetes mellitus in association with CIMT (Karim et al. 2005). To clarify the relevance of lipid status, it would be interesting to investigate our hypothesis among cohorts with familial hypercholesteremia (Wiegman et al. 2004; Wittekoek et al. 1999).

As shown in Figure 3, the size of the point estimate was larger among the older subjects. Future research needs to clarify whether air pollution contributes to atherosclerosis only after a certain age or early on. Effects of air pollution on lung development have been observed during adolescence and may be a result of both pulmonary and chronic systemic inflammatory effects (Gauderman et al. 2002); thus, it is conceivable that atherogenic responses may occur early in life. The age dependence of the effects may also be codetermined by genetic factors (Humphries and Morgan 2004; Ross 1999).

We also observed larger effects in women. If other cardiovascular risk factors such as occupational exposures dominate atherosclerosis in men, we would expect a smaller effect signal and less precision in the estimates among men. We also hypothesize that interactions may reflect biologic causes. If premenopausal women are protected against atherosclerosis by endogenous hormones, loss of hormonal protection would lead to increased vulnerability after menopause (Kannel et al. 1976). This could explain part of the interaction by both age and sex.

Active and passive smoking did not confound results in either the total sample or among subgroups. Adjustment for active tobacco smoke led to a slight increase in the effect estimate; thus, residual confounding is unlikely to overestimate the effects. However, PM_2.5_ associations were clearly stronger in never smokers compared with smokers (data not shown). This gradient was also observed in all subgroups with significant PM_2.5_ associations (Figure 3). Oxidative and inflammatory effects of smoking may dominate to such an extent that the additional exposure to ambient air pollutants may not further enhance effects along the same pathways. The difference in the effects of PM_2.5_ in smokers and nonsmokers needs further investigation. The American Cancer Society cohort study does not reveal a clear pattern of a smoking interaction for the association of ambient air pollution and cardiovascular death (Krewski et al. 2004; Pope et al. 2004). In the Study on Air Pollution and Lung Diseases in Adults (SAPALDIA), associations between air pollution and level of pulmonary function did not differ by smoking status (Ackermann-Liebrich et al. 1997).

Some U.S. studies indicate effect modification of air pollution by socioeconomic status (SES) with much stronger effects among the less educated (Pope et al. 2002). The cause of this interaction pattern is not well understood. SES status was rather homogeneous in these mostly well-educated volunteers, providing little power to investigate interactions of pollution with SES. If lower SES also positively modifies effects of air pollution on atherosclerosis, our population would provide an underestimate of the health effects in the general population (O’Neill et al. 2003). Further research on samples representative of the population will be needed to assess whether the high SES in the clinical trials biases the effects toward the null.

Future research should focus on identifying factors that determine susceptibility to PM_2.5_. We are initiating studies on subjects with inflammatory metabolic syndromes prone to accelerated atherosclerosis such as postmenopausal women, diabetics, or obese or physically inactive people. To corroborate the cross-sectional findings, follow-up studies are ultimately needed to investigate the association of concurrent levels of air pollution exposure with the progression of CIMT.

### Plausibility

From a biologic perspective, our results support the hypothesis that long-term exposure to ambient PM contributes to systemic inflammatory pathways, which are a relevant aspect of atherogenesis (Ross 1999). The findings indicate a biologically plausible link between the observed acute effects of ambient air pollution on systemic inflammation (Glantz 2002) and the long-term consequences of sustained vascular inflammation leading to increased atherosclerosis and, ultimately, cardiovascular death (Hoek et al. 2002; Pope et al. 2004). Among susceptible people, this may lead to artery wall lesions similar to those observed in the rabbit model (Fujii et al. 2002; Suwa et al. 2002). In these hyperlipidemic rabbits, 4-week PM exposure was associated with the progression of atherosclerotic lesions, coupled with an enhanced release of bone marrow monocytes. These precursors of macrophages play an important role in the atherogenic inflammatory responses (Goto et al. 2004; Ross 1999; Suwa et al. 2002). Given the central role of oxidized LDL in the initiation and progression of atherogenesis, suggestions that the plasma of automotive workers with high exposure to traffic exhaust is more susceptible to oxidation is also of interest (Sharman et al. 2002).

As a quantitative plausibility check, we compared the size of the PM_2.5_ effects with effects of other risk factors on CIMT. Using smoking and environmental tobacco smoke (ETS) as a model for air pollution exposure, the size of our estimates appear plausible (Diez-Roux et al. 1995; Howard et al. 1994). Associations of ETS and current levels of air pollution with various respiratory outcomes are similar and support the notion of common underlying pathways (Künzli 2002). Smoking and ETS associate with stiffer and thicker artery walls, reflecting the systemic effect of these exposures (Howard et al. 1994; Mack et al. 2003). Exposure to ETS was associated with 2–3% thicker intima-media, which approximate the effects observed for a 10 μg/m3 change in PM_2.5_ (Diez-Roux et al. 1995; Howard et al. 1994). Using never smokers without ETS exposure as the referent group in our data, never smokers with ETS at home had 0.9% (–2.7 to 4.5%) thicker artery walls; former smokers’ CIMT was increased on average by 3.4% (0.7–6.3%), and the 30 current smokers had 5% (–1.5 to 11.6%) thicker CIMT. The trend across these four categories of tobacco exposure was statistically significant. As shown in Table 1, smokers were underrepresented in these volunteers of well-educated participants.

The observed percent change in CIMT corresponds to an increase in the thickness of approximately 20–40 μm per 10 μg/m^3^ contrast in PM_2.5_. This difference in CIMT translates into some 3–6% increase in the long-term risk for myocardial infarction (O’Leary et al. 1999). Pope et al. (2004) reported that long-term exposure to PM_2.5_ was associated with an 18% (14–23%) increase in ischemic heart disease. Effect sizes reported here concur with these findings, indicating that a fraction of the total effect of ambient PM on cardiovascular mortality may be mediated through sustained long-term effects of air pollution on atherosclerosis (Künzli et al. 2001). This is in line with the proposed model (Künzli et al. 2001) in which some of the effects observed in cohort studies must reflect long-term contributions of air pollution to the underlying disease progression, whereas in other cases, air pollution contributes only to triggering of cardiovascular events or death (Bell et al. 2004; Künzli et al. 2001; Peters and Pope 2002).

From a biologic and policy perspective, we emphasize that PM_2.5_ probably serves as a surrogate for the mixture of urban air pollution and constituents of PM. It is premature to conclude that PM_2.5_ and its constituents are the atherogenic culprit per se. Atherosclerosis results from complex processes that may include a combination of various urban pollutants, host factors, and pathways that ultimately lead to the findings of a CIMT–PM_2.5_ association.

In conclusion, we have presented the first epidemiologic evidence supporting the idea of a chronic vascular response to respiratory and systemic effects of PM exposure. Given the leading role of heart disease as a cause of death in most westernized countries and the growing contribution in developing countries, these findings may be of high public health relevance. Further investigations need to focus on susceptible groups and follow-up of cohorts to investigate the effect of air pollution on the progression of CIMT.

## Figures and Tables

**Table 1:** Description of assigned exposure (outdoor concentration in 2000) and CIMT, and main characteristics of the study population at the time of baseline measurements in the total sample, men, women, women ≥60 years of age, and subjects under lipid-lowering therapy.

Characteristic	Total sample (*n* = 798)	Males (*n* = 443)	Females (*n* = 355)	Females ≥60 years (*n* = 186)	Lipid-lowering therapy (*n* = 109)
PM_2.5_ (μg/m^3^)	20.3 ± 2.6	20.1 ± 2.7	20.5 ± 2.4	20.7 ± 2.3	20.0 ± 2.5
Ozone (ppb, annual mean of daily maximum)	89.2 ± 17.9	89.6 ± 18.5	88.8 ± 17.3	87.1 ± 17.2	88.5 ± 18.6
CIMT (μm)	755 ± 148	767 ± 166	740 ± 118	775 ± 120	788 ± 140
Age (years)	59.2 ± 9.8	58.3 ± 10.3	60.4 ± 8.9	67.3 ± 5.3	63.3 ± 10.0
Diastolic blood pressure (mm Hg)	77.8 ± 9.2	79.2 ± 8.8	75.9 ± 9.3	74.8 ± 9.5	78.1 ± 8.9
Systolic blood pressure (mm Hg)	127.2 ± 16.3	126.7 ± 16.0	127.8 ± 16.6	130.5 ± 16.7	130.9 ± 16.2
LDL cholesterol (mg/dL)	137.9 ± 29.5	137.0 ± 30.9	139.0 ± 27.6	136.4 ± 26.9	125.7 ± 33.7
White (%)	67.3	67.7	66.8	65.0	69.7
Smoking status (%)					
Never smokers	62.9	62.8	63.1	62.9	53.2
Former smokers	33.2	33.4	33.0	33.3	44.0
Current smokers	3.8	3.6	3.9	3.8	2.8
ETS at home (%)	33.5	21.9	47.9	55.4	37.5
Lipid-lowering therapy (%)	13.7	15.3	11.5	15.1	100
Antihypertensive prescriptions (%)	26.2	26.6	25.6	33.3	42.2

ETS, environmental tobacco smoke. Data are mean ± SD except where indicated.

**Table 2:** Percent change (and 95% CI) in CIMT (μm) associated with a 10 μg/m^3^ change in ambient outdoor PM_2.5_ concentration at the residential ZIP code in the total population (*n* = 798).*^a^*

	Total sample (798)		Females ≥60 years (186)		Lipid-lowering therapy (109)	
Model*^a^* (with adjustment factors in the model)	Percent change	*p*-Value	Percent change	*p*-Value	Percent change	*p*-Value
None (unadjusted estimate)	5.9 (1.0–10.9)	0.018	19.2 (8.8–30.5)	0.001	15.8 (2.1–31.2)	0.024
Age, sex, education, income*^b^*	4.4 (0.0–9.0)	0.056	15.7 (5.7–26.6)	0.002	13.3 (0–28.5)	0.051
All above plus active and passive smoking, multivitamins, alcohol	4.2 (^–^0.2–8.9)	0.064	13.8 (4.0–24.5)	0.002	13.3 (^–^0.3–28.8)	0.060

a Unadjusted association (crude model) and estimates from two multivariate models; 95% CIs of the estimates are shown in parentheses. The relative effects are based on a linear model with log intima-media thickness as dependent variable.

b Factors with univariate associations with both, CIMT and PM_2.5_.
